# Heavy metal contamination in Peru: implications on children’s health

**DOI:** 10.1038/s41598-021-02163-9

**Published:** 2021-11-23

**Authors:** Xulia Fandiño Piñeiro, Mauro T. Ave, Narmeen Mallah, Francisco Caamaño-Isorna, A. Nuria Guisández Jiménez, Duarte Nuno Vieira, Flaviano Bianchini, José Ignacio Muñoz-Barús

**Affiliations:** 1 Department of Forensic Sciences, Pathology, Gynecology and Obstetrics, Pediatrics, University of Santiago de Compostela, Santiago de Compostela, Spain; 2 Institute of Forensic Sciences, University of Santiago de Compostela, Santiago de Compostela, Spain; 3 Complejo Hospitalario Universitario de Santiago de Compostela, Santiago de Compostela, Spain; 4 Centro de Investigación Biomédica en Red de Enfermedades Cardiovasculares (CIBERCV), Madrid, Spain; 5 Instituto de Investigación Sanitaria de Santiago (IDIS), Santiago de Compostela, Spain; 6 Department of Preventive Medicine, University of Santiago de Compostela, R/San Francisco, s/n, 15782 Santiago de Compostela, Spain; 7 Centro de Investigación Biomédica en Red de Epidemiología y Salud Pública (CIBERESP), 28029 Madrid, Spain; 8 Department of Legal Medicine and Ethical Sciences, Faculty of Medicine, University of Coimbra, Coimbra, Portugal; 9 Source International, Calci, Pisa, Italy

**Keywords:** Environmental impact, Environmental sciences, Risk factors

## Abstract

Cerro de Pasco, Peru, has been excessively contaminated with heavy metals due to high mining activities in the region. We investigated the presence of chronic exposure to heavy metals in children living in Cerro de Pasco and its effect on health. Heavy metal concentrations were determined in hair samples collected from 78 children living in a region exposed to an open-pit mine (Paragsha region) and from other 16 children unexposed to mine activities (Carhuamayo region). Children exposed to the mine showed statistically significant higher concentration of aluminum, antimony, arsenic, cadmium, chromium, iron, lead, tin and thallium (p < 0.05) than control children. Hair samples collected from the same children in two occasions (2016 and 2018) showed that the exposure is chronic with higher levels of heavy metals observed in 2018. The concentration of heavy metals was higher in hair tip than in hair root samples. Heavy metals are associated with substantial higher risk of nosebleed (odds ratio, OR = 15.40), chronic colic (OR = 7.30), dermatologic alterations (OR = 6.16), mood alterations (OR = 7.07), presence of white lines on nails (OR = 12.10), reduced visual camp (OR = 3.97) and other symptoms (OR = 5.12). Chronic heavy metal exposure implies various negative consequences on children’s health. Preventive measures are crucial to protect children’s health.

## Introduction

Heavy metals is a widely used term to refer to a category of metallic elements of high molecular weight (≥ 20) and density when compared to water (≥ 5 g/cm^3^)^1,2^. Heavy metals are naturally occurring, and their presence at very low levels is essential to maintain various biochemical and physiological functions in living organisms^3^.

Human activities such as mining contribute to increasing the release of heavy metals into the environment^4^. Contamination caused by mining activities is associated with huge environmental burdens, and is considered as a situation of environmental crime by many experts. Environmental crimes are characterized by their impact on the nature, increasing the levels of pollution, degrading the wildlife, reducing the biodiversity, disturbing the ecological balance and causing irreversible climate change^5^. Several analytical methods to detect heavy metals in food chain and environment and to eliminate those metals when present in high concentrations have been published elsewhere^6–10^.

Exposure to high levels of heavy metals is also associated with health consequences, though their toxic effects vary according to the forms and routes of exposure^11^. Heavy metals usually interrupt intracellular homeostasis through the production of free radicals which interact with other chemicals and biological molecules and consequently lead to lipid, protein and DNA damage^11^. For instance, arsenic, a highly toxic heavy metal, stimulates the production of arsenic induced superoxide which plays a main role in disrupting various cell signalling pathways through the interaction with biological macromolecules, leading to DNA damage, lipid peroxidation and alteration of antioxidant enzyme levels^12^. Consequently, heavy metals exposure contributes to the development of major diseases such as cancer and respiratory, neurological, and kidney diseases, and eventually drives to death^1,3,13–16^.

In Peru, the mining sector has been continuously expanding with the mining activities concentrated in the central and southern regions of the country^17^. Peru ranks among the top five producers of heavy metals like copper, lead and zinc^18^. The City of Cerro de Pasco, located at the top of Peruvian Andes, is a mining town and considered one of the most contaminated places on earth^19^. The mining activity in the Peruvian Andes takes place on farmers community lands and headwaters^20^, and the presence of excessive levels of heavy metals in the air, soil (agricultural and recreational) and water has been already demonstrated^20–25^. More than one and a half million people in Peru are exposed to lead in soil^26^. The Peruvian government has recently placed a barrier surrounding the mine to reduce heavy metal pollution.

A recent report has pointed out the devastating impact of heavy metal contamination due to metallurgical emissions on health in Cerro de Pasco^27^. Previous studies also reported alarming figures such as the presence of blood-lead levels higher than 10 µg/dL in children living in this district^21,23–25^. Lead is classified as carcinogenic and is the second most harmful heavy metal after arsenic^28^. Though such disturbing findings have been reported since 1996^29^, the health consequences of heavy metal pollution in Peru have not been sufficiently investigated, standards to regulate the heavy metal content such as lead in the soil are still unavailable^26^, and regulations to control mining activities are weak.

Accordingly, we aimed in the present study to determine the health effects of exposure to heavy metals in a children population in Cerro de Pasco (Peru), and to investigate if the barrier placed around the mine reduces the exposure to heavy metals. We also explored the association of chronic pollution with certain health conditions and examined the effect of exposure to multiple heavy metals on body organs and systems.

## Materials and methods

### Settings and population

Cerro de Pasco is the capital of the province of Pasco, located in the Central Zone of Perú and has a population size of around 80,000 inhabitants. An open pit mine is located in the same capital, and the mining activities generates air, water and soil contamination^21,23^.

The study population consisted of children between 3 and 16 years of age who had a permanent residence in the study area: Paragsha zone in Cerro de Pasco and Carhuamayo city. The open-air mine hole is situated at approximately 400 m from Paragsha zone, i.e., Paragsha is directly exposed to the activities carried out in the mine. Carhuamayo is located at 35 km from Cerro de Pasco in a southeast direction but shares similar socio-geographic and atmospheric characteristics to those of Paragsha.

### Ethics

The participation in the study was voluntary. Parents of participating children provided a signed informed consent before their recruitment. The study was approved by the ethics committee of the University of Santiago de Compostela (Protocol No. 08102019) and was carried out in compliance with the corresponding legal and ethical requirements (Spanish Acts 14/2007 and 3/2018).

### Sample

In 2018, hair samples were collected from 94 children. Hair was the type of chosen samples to measure heavy metal concentration because it allows determining if the children have been chronically exposed to these metals, and at the same time it is also a non-invasive and stable method^30^. Due to the lack of standards that specify the “normal” and the “toxic” levels of heavy metals in hair, and in line with previous studies, we adopted the reference values for children that were established by the Micro Trace Minerals (ER) laboratory (Table 1)^23^.

**Table 1:** Average concentrations of heavy metals measured in hair roots and hair tip samples collected in 2018 from children exposed and unexposed to the mine.

Metal (mg/kg)	Reference value^a^	Exposed group (Paragsha)		Unexposed group (Carhuamayo)	
		Hair root	Hair tip	Hair root	Hair tip
Al	< 8.00	30.22	47.30	22.31	26.90
Sb	< 0.20	0.12	0.24	0.05	0.14
As	< 0.20	0.47	0.42	0.18	0.14
Ba	< 2.65	1.18	4.98	0.86	3.72
Be	< 0.03	0.002	0.005	0.001	0.003
B	< 0.84	2.64	2.70	1.72	2.19
Cd	< 0.20	0.10	0.40	0.04	0.13
Co	< 0.15	0.04	0.17	0.02	0.074
Cr	0.02–0.15	0.83	1.61	0.39	0.88
Fe	7.70–15.00	55.11	68.36	29.82	42.30
Mn	0.07–0.50	5.22	13.41	2.15	4.74
Hg	< 0.30	0.24	0.41	0.34	0.31
Mo	0.02–1.00	0.24	0.20	0.80	0.09
Ni	< 0.85	1.96	9.78	2.96	14.11
Pb	< 0.10	4.58	10.37	0.81	2.69
Cu	6.70–37.00	13.49	27.27	10.46	17.60
Se	0.40–1.70	8.73	1.26	0.79	0.71
Sn	0.93	42.0	79.16	0.11	0.55
Tl	< 0.01	0.02	0.02	0.01	0.005
V	0.01–0.15	0.06	0.20	0.04	0.08
Zn	110.0–227.0	200.71	488.79	188.81	258.00

^a^Reference values for children used by Micro Trace Minerals Laboratory in Germany. Hair tip samples were collected at 20 cm from the hair root.

*Al* aluminium, *As* arsenic, *B* boron, *Ba* barium, *Be* beryllium, *Cd* cadmium, *Co* cobalt, *Cr* chromium, *Fe* iron, *Mn* magnesium, *Hg* mercury, *Mo* molybdenum, *Ni* nickel, *Pb* lead, *Cu* copper, *Sb* antimony, *Se* selenium, *Sn* tin, *Tl* thallium, *V* vanadium, *Zn* zinc.

Seventy-eight children were recruited from Paragsha; the region exposed to heavy metals. Twenty-seven of the 78 exposed children were included in a previous study that was undertaken in 2016^23^, hence data from two periods are available for those children. Sixteen other children were recruited from Carhuamayo and served as the group unexposed to heavy metals.

### Anamnesis and examination

All recruited children underwent a complete physical examination and anamnesis. In the anamnesis, we inquired about respiratory, digestive, bleeding and neurological antecedents, among others. In addition, data were collected on vaccine administration, medical and hospitalization history and the reasons for medical assistance or interventions, when applicable.

In the clinical examination, we assessed many ophthalmoscopic and otoscopic aspects such as: children's development and general condition, as well as abdominal, respiratory, dermatological, ophthalmological, cardiovascular, digestive, auditory, urinary and neurological complaints. Regarding the external examination, information was collected on the presence/absence of skin with an earthy color, breath and acid sweat, bleeding, fever at the time of the evaluation, among others. The presence of specific features potentially related to exposure to heavy metals was investigated, such as: Café-au-lait spots (coffee-with-milk-colored spots) in various parts of the children's body, deafness, inflamed conjunctiva, bluish lines on the edge of the gums, constipation, diarrhea, cramps and white lines on the nails or Mees lines.

At the end of the examination, hair samples were collected from the nape of the neck.

### Laboratory analysis

All the collected hair samples were processed, using 1 cm fragments, and analyzed, in a certified laboratory, by Inductively Coupled Plasma Mass Spectrometry, ICP-MS, following previously published procedures^31^. The concentration of 21 heavy metals was determined: aluminium (Al), antimony (Sb), arsenic (As), boron (B), barium (Ba), beryllium (Be), cadmium (Cd), cobalt (Co), chromium (Cr), iron (Fe), magnesium (Mn), mercury (Hg), molybdenum (Mb), nickel (Ni), lead (Pb), copper (Cu), selenium (Se), tin (Sn), thallium (Tl), vanadium (V) and zinc (Zn).

The analytical procedure consisted of the following steps: (1) cutting the hair samples into parts of about 1 cm, (2) immersion of the hair samples in a mixture of acetone/ethyl ether (1 + 4 v/v) for 30 min, (3) drying in a stove at a maximum of 40 °C, (4) second immersion in 5% ethylenediaminetetraacetic acid (EDTA) solution for 30 min, (5) rinsing with de-ionized water, (6) drying in a stove at a maximum of 40 °C, (7) after the washing phase, each sample was weighed (about 0.1000—0.4000 g) and subsequently mineralized in a microwave device in high-pressure Teflon vessels, with a mixture of nitric acid and hydrogen peroxide in proportion 5 + 1 (v/v). The thermal program consisted of two steps. In the first step, the samples were incubated at 90 °C for 3 min. In the second step, the samples were placed at 120 °C for 5 min. Subsequently, the samples were cooled down for 30 min. A blank control and a reference material were also mineralized with each sequence of samples. The solution was transferred into a volumetric flask and analyzed by ICP-MS technique.

Results were sub-grouped in order to compare the part of the hair that has been exposed to metals the longest (hair tip, at a 20 cm from the hair root) to the part of the hair that has been exposed for the least duration (hair root). Assuming that the rate of hair growth is 0.6–1.4 cm / month, a hair tip located at 20 cm from the root, would be equivalent to an exposure duration of 12 to 28 months before sample collection^32^.

### Statistical analysis

#### Chronic exposure to heavy metals

To determine the presence of chronic exposure to heavy metals, we calculated the mean difference of heavy metal concentration between hair root samples of the same individuals collected in 2016 and 2018 and tested the statistical significance of this difference using T-student test.

T-student test for paired samples was also undertaken to compare heavy metal concentrations in hair tip samples collected in 2018 with that of hair root of the same individuals (Table S2 of supplementary materials).

#### Association of heavy metals with clinical symptoms

We investigated the associations of heavy metal concentrations, irrespective of the place of residence, with the manifestation of clinical symptoms (nosebleed, white line on nails and chronic colic) by calculating Pearson’s chi-squared X^2^ test p-value. We also estimated X^2^ P-value of linear association to explore if association between heavy metals and clinical symptoms follows a linear trend. For the purpose of these analyses, children were sub-grouped into terciles of the concentration of heavy metals detected in their hair: low, medium, and high.

To verify if the manifestation of nosebleed and the presence of white lines on nails is related to exposure to heavy metals, data from a mixed population composed of exposed and unexposed children to the mine were compared using T-student test for independent samples (see supplementary material online, Table S3).

#### Association of the place of residence with heavy metal concentration and clinical manifestation

The association of heavy metal concentration with the place of residence (Carhuamayo versus Paragsha) was examined using T-student test. The association of the place of residence with the presence of clinical symptoms was determined by estimating the odds ratio (OR) using logistic regression models.

Analyses were carried out using SPSS (SPSS Inc. Released 2011. SPSS for Windows, Version 20.0. Chicago.

## Results

### Participants’ general characteristics

Seventy-eight exposed and 16 unexposed children to the mine participated in the study. The gender ratio of the exposed children was 1:1 (39 boys and 39 girls). Three-quarters of the unexposed population were females (N = 12). Both groups, exposed and unexposed, were similar in terms of age (exposed children: average = 10.01 years; range: 3–16 and unexposed children: average = 10 years; range: 5–15), weight (exposed children: average = 31.60 kg; range: 9–60 and unexposed children: average = 31.16 kg; range: 17–46), height (exposed children: average = 132.68 cm; range: 95–164 and unexposed children: average = 134.62 cm; range: 111–152), body temperature (exposed children: average = 35.93 °C; range: 34.10–37.70 and unexposed children: average = 35.53 °C; range: 33.4–36.0 °C), systolic blood pressure (exposed children: average = 86.28 mmHg; range: 70–120 and unexposed children: average = 82.13 mmHg; range: 65–105 mmHg), diastolic blood pressure (exposed children: average = 54.68 mmHg; range: 38–85 mmHg and unexposed children: average = 51.88 mmHg; range: 38–70 mmHg), and pulse rate [exposed children: average = 76.90 pulse per minute (ppm); range: 48–108 and unexposed children: average = 79.29 ppm; range: 64–108].

In the population exposed to the mine (Paragsha region), the average concentration of heavy metals significantly differed between male and female children (see supplementary material online, Table S1). Due to the limited number of male children in the non-exposed population, gender differences with regards to heavy metals concentration were not explored.

### Presence of heavy metals in hair root and hair tip

Table 1 represents the average concentration of heavy metals measured in hair root and hair tip (at 20 cm from hair root samples) samples collected in 2018 from children living in Paragsha (exposed group) and Carhuamayo (unexposed group). In reference to ER, children exposed to the mine had high concentrations of Al, As, B, Cr, Fe, Mn, Ni, Pb, Se, Sn and Tl in their hair root and of Al, Sb, As, Ba, B, Cd, Co, Cr, Fe, Mn, Hg, Ni, Pb, Sn, Tl, V and Zn in their hair tip. Children unexposed to the mine had elevated concentrations of Al, B, Cr, Fe, Mn, Hg, Ni and Pb in their hair root as well as in their hair tip (Table 1).

### Chronic exposure to heavy metals

The comparison of heavy metal concentrations between hair root samples collected in 2016 and 2018 revealed a chronic exposure. Hair root samples collected in 2018 have statistically significant higher concentrations of Al, Sb, Ba, Cd, Co, Cr, Fe, Mn, Mo, Ni, Pb, Cu, Se, Sn, V and Zn than that in samples collected in 2016 (Table 2). The largest differences of concentrations were observed for Sn (difference of average concentration between 2018 and 2016 = 30.98 mg/Kg) and Al (difference of average concentration between 2018 and 2016 = 10.17 mg/Kg). Inversely, the heavy metals B and Be were more abundant in samples collected in 2016 than that of 2018.

**Table 2:** Comparison of the mean concentrations of heavy metals detected in hair root samples collected in 2016 and 2018 from the same individuals living in Paragsha.

Metal	Concentration (mg/kg)		Mean difference (mg/kg)	T-test P-value
	2016	2018		
Al	18.85	29.02	−10.17	0.01
B	4.48	2.08	2.40	< 0.001
Ba	0.56	1.09	−0.53	< 0.001
Be	0.02	0.00	0.02	< 0.001
Cd	0.04	0.11	−0.08	< 0.001
Co	0.02	0.04	−0.03	0.016
Cr	0.26	0.71	−0.45	< 0.001
Fe	24.09	52.85	−28.76	< 0.001
Mn	1.82	4.93	−3.11	< 0.001
Mo	0.06	0.08	−0.03	0.002
Ni	0.19	1.47	−1.28	< 0.001
Pb	2.89	4.26	−1.37	0.039
Cu	7.99	11.30	−3.31	< 0.001
Sb	0.06	0.12	−0.06	0.002
Se	0.67	0.86	−0.18	< 0.001
Sn	0.12	31.10	−30.98	< 0.001
V	0.03	0.05	−0.02	< 0.001
Zn	129.11	206.85	−77.74	< 0.001

*Al* aluminium, *B* boron, *Ba* barium, *Be* beryllium, *Cd* cadmium, *Co* cobalt, *Cr* chromium, *Fe* iron, *Mn* magnesium, *Mo* molybdenum, *Ni* nickel, *Pb* lead, *Cu* copper, *Sb* antimony, *Se* selenium, *Sn* tin, *V* vanadium, *Zn* zinc.

The concentration of the tested heavy metals significantly differed between hair tip and hair root in samples collected in 2018 from the same individuals (T-test p-value > 0.05) (see supplementary material online, Table S2). Hair tip samples showed higher concentrations of Al, Sb, Ba, Be, Cd, Co, Cr, Fe, Mn, Mo, Pb, Cu, Sn, Tl, V and Zn than hair root samples.

### Association of heavy metals with the presence of certain clinical symptoms

A statistically significant association was observed between exposure to heavy metals and the presence of specific clinical signs (T-student for independent samples p-value < 0.05) (see supplementary material online, Table S3). We observed statistically significant associations between Al, As, Cd, Cr, Fe, Mn, Pb, Sn and Tl and nose bleeding; between Al, Cd, Fe, Mn, Tl and V and the presence of white lines on the nails; and between As and B and chronic colic.

### Association of heavy metals with clinical manifestations

There is a linear association between high heavy metal concentrations and the presence of specific clinical signs, irrespective of the place of residence (p-value < 0.05) (Table 3). In specific, high level of As, Cd, Fe, Mn, Pb and Tl are associated with a history of nosebleed. The heavy metals Al, Sb, As, Cd, Co, Cr, Fe, Mn, Pb, Sn, Tl and V are associated with the presence of white lines on the nails. As and B are associated with chronic colic.

**Table 3:** Relation between heavy metal concentration and the presence of nosebleed, white lines on nails and chronic colic.

Metal	Concentration	Nosebleed				White lines on nails				Chronic colic			
		No N (%)	Yes N (%)	X^2^ p-value		No N (%)	Yes N (%)	X^2^ p-value		No N (%)	Yes N (%)	X^2^ p-value	
				linear association	Pearson			linear association	Pearson			Linear association	Pearson
As	Low	21 (67.7)	10 (32.3)	0.001	0.01	27 (87.1)	4 (12.9)	0 .039	0.07	26 (83.9)	5 (16.1)	0.045	0.1
	Medium	13 (41.9)	18 (58.1)			20 (64.5)	11 (35.5)			19 (61.3)	12 (38.7)		
	High	8 (26.7)	22 (73.3)			19 (63.3)	11 (36.7)			18 (60.0)	12 (40.0)		
Cd	Low	21 (67.7)	10 (32.3)	< 0.001	< 0.001	30 (96.8)	1 (3.2)	0.001	< 0.001	23 (74.2)	8 (25.8)	0.277	0.5
	Medium	14 (46.7)	16 (53.3)			18 (60.0)	12 (40.0)			21 (70.0)	9 (30.0)		
	High	7 (22.6)	24 (77.4)			18 (58.1)	13 (41.9)			19 (61.3)	12 (38.7)		
Fe	Low	22 (73.3)	8 (26.7)	0.001	< 0.001	27 (90.0)	3 (10.0)	< 0.001	< 0.001	23 (76.6)	7 (23.3)	0.199	0.4
	Medium	10 (32.3)	21 (67.7)			24 (77.4)	7 (22.6)			21 (67.7)	10 (32.3)		
	High	10 (32.3)	21 (67.7)			15 (48.4)	16 (51.6)			19 (62. 3)	12 (38.7)		
Mn	Low	21 (70.0)	9 (30.0)	0.045	< 0.001	27 (90.0)	3 (10.0)	0.008	0.02	24 (80.0)	6 (20.0)	0.519	0.1
	Medium	7 (23.3)	23 (76.7)			20 (66.7)	10 (33.3)			16 (53.3)	14 (46.7)		
	High	14 (43.8)	18 (56.2)			19 (59.4)	13 (40.6)			23 (71.9)	9 (28.1)		
Pb	Low	21 (70.0)	9 (30.0)	0.001	< 0.001	27 (90.0)	3 (10.0)	0.002	0.01	24 (80.0)	6 (20.0)	0.168	0.2
	Medium	12 (38.7)	19 (61.3)			22 (71.0)	9 (29.0)			19 (61.3)	12 (38.7)		
	High	8 (26.7)	22 (73.3)			16 (53.3)	14 (46.7)			19 (63.3)	11 (36.7)		
Tl	Low	22 (73.3)	8 (26.7)	0.001	< 0.001	< 0.001	3 (10.0)	0.001	0	25 (83.3)	5 (16.7)	0.057	0.1
	Medium	10 (34.5)	19 (65.5)			< 0.001	7 (24.1)			18 (62.1)	11 (37.9)		
	High	10 (30.3)	23 (69.7)			< 0.001	16 (48.5)			20 (60.6)	13 (39.4)		
Al	Low	Not measured				29 (90.6)	3 (9.4)	0.001	< 0.01	23 (71.9)	9 (28.1)	0.789	0.8
	Medium					20 (71.4)	8 (28.6)			18 (64.3)	10 (35.7)		
	High					17 (53.1)	15 (46.9)			22 (68.8)	10 (31.2)		
Sb	Low	Not measured				28 (96.6)	1 (3.4)	< 0.001	< 0.01	23 (79.3)	6 (20.7)	0.352	0.2
	Medium					22 (68.8)	10 (31.2)			19 (59.4)	13 (40.6)		
	High					16 (51.6)	15 (48.4)			21 (67.7)	10 (32.3)		
Co	Low	Not measured				30 (93.8)	2 (6.2)	0.003	< 0.01	26 (81.2)	6 (18.8)	0.208	0.1
	Medium					18 (60.0)	12 (40.0)			17 (56.7)	13 (43.3)		
	High					18 (60.0)	12 (40.0)			20 (66.7)	10 (33.3)		
Cr	Low	Not measured				26 (89.7)	3 (10.3)	0.002	0.01	21 (72.4)	8 (27.6)	0.402	0.7
	Medium					23 (74.2)	8 (25.8)			22 (71.0)	9 (29.0)		
	High					17 (53.1)	15 (46.9)			20 (62.5)	12 (37.5)		
Sn	Low	Not measured				24 (85.7)	4 (14.3)	0.019	0.06	22 (78.6)	5 (21.4)	0.258	0.4
	Medium					24 (72.7)	9 (27.3)			21 (63.6)	12 (36.4)		
	High					18 (58.1)	13 (41.9)			20 (64.5)	11 (35.5)		
V	Low	Not measured				26 (89.7)	3 (10.3)	0.006	0.02	23 (79.3)	6 (20.7)	0.197	0.3
	Medium					21 (70.0)	9 (30.0)			19 (63.3)	11 (36.7)		
	High					19 (57.6)	14 (42.4)			21 (63.6)	12 (36.4)		
B	Low	Not measured				19 (70.4)	8 (29.6)	0.842	0.98	24 (88.9)	3 (11.1)	0.23	< 0.001
	Medium					23 (71.9)	9 (28.1)			20 (62.5)	12 (37.5)		
	High					24 (72.7)	9 (27.3)			19 (57.6)	14 (48.3)		

*N* number of children, *X*^*2*^ chi-square test, *Al* aluminium, *As* arsenic, *B* boron, *Cd* cadmium, *Co* cobalt, *Cr* chromium, *Fe* iron, *Mn* magnesium, *Pb* lead, *Sb* antimony, *Sn* tin, *Tl* thallium, *V* vanadium.

### Association of area of residence with heavy metal concentration

There is a statistically significant association between the residence area and the concentration of the following heavy metals: Al, Sb, As, Cd, Cr, Fe, Pb, Sn and Tl (Table 4). The concentration of these metals in the hair root of children exposed to the mine is substantially higher than that determined in the hair root of unexposed children. The most notable differences were observed for tin (Sn) [average concentration in Carhuamayo (non-exposed region) = 0.11 mg/Kg and in Paragsha (exposed region) = 42.00 mg/Kg] and lead (Pb) [average concentration in Carhuamayo = 0.81 mg/Kg and in Paragsha = 4.62 mg/Kg].

### Association of area of residence with specific clinical signs

#### Nosebleed

Half of the children exposed to the mine frequently suffered from nosebleed, while only 6.2% of the unexposed children reported a history of nosebleed. The odds of nosebleed are 15 times higher for exposed than that for unexposed children (OR = 15.45, p-value < 0.001) (Table 5).

**Table 4:** Association between area of residence and heavy metal concentration.

Metal (mg/Kg)	Residence area	N	Average concentration (mg/kg)	T-student p-value
Al	Carhuamayo	16	22.3125	0.025
	Paragsha	76	30.2171	
Sb	Carhuamayo	16	0.0498	< 0.001
	Paragsha	76	0.1156	
As	Carhuamayo	16	0.1762	< 0.001
	Paragsha	76	0.4676	
Cd	Carhuamayo	16	0.0416	0.001
	Paragsha	76	0.0988	
Cr	Carhuamayo	16	0.3931	< 0.001
	Paragsha	76	0.8303	
Fe	Carhuamayo	16	29.8157	< 0.001
	Paragsha	76	55.1053	
Pb	Carhuamayo	16	0.8100	< 0.001
	Paragsha	76	4.6247	
Sn	Carhuamayo	16	0.1139	< 0.001
	Paragsha	76	41.9969	
Tl	Carhuamayo	16	0.0051	< 0.001
	Paragsha	76	0.192	

Carhuamayo: region unexposed to the mine; Paragsha: region exposed to the mine.

*N* number of children.

**Table 5:** Association between place of residence and manifestation of certain clinical signs.

Clinical sign	Unexposed region (Carhuamayo) N (%)	Exposed region (Paragsha) N (%)	Odds ratio	Odds ratio p-value
**History of nosebleed**				
No	14 (87.5)	29 (37.2)	15.40	< 0.001
Yes	2 (12.5)	49 (62.8)		
**White lines on nails**				
No	16 (100)	51 (65.4)	12.10	0.001
Yes	0 (0.0)	27 (34.6)		
**Chronic colic**				
No	15 (93.8)	49 (62.8)	7.30	0.007
Yes	1 (6.2)	29 (37.2)		
**Dermatologic alterations**				
No	16 (100)	63 (80.8)	6.16	0.013
Yes	0 (0)	15 (19.2)		
**Mood alterations**				
No	16 (100)	61 (78.2)	7.07	0.008
Yes	0 (0)	17 (21.8)		
**Constipation**				
No	16 (100)	64 (82.1)	5.71	0.017
Yes	0 (0)	14 (17.9)		
**Reduced visual field**				
No	16 (100)	68 (97.2)	3.97	0.046
Yes	0 (0)	10 (12.8)		
**Other clinical symptoms**				
No	15 (93.8)	54 (69.2)	5.12	0.024
Yes	1 (6.2)	24 (30.8)		

*N* number of children, *X*^*2*^ chi-square test.

Dermatologic alterations include café-au-lait spots in flexor areas of the abdomen, back, chest or neck, calluses on palms of the hands, soles with thick and rough skin and calluses in areas without friction; other clinical symptoms encompassed bluish line on the gum, calluses in body areas without friction, calluses on palms and sole with thick and rough skin and inflamed conjunctiva.

#### White lines on nails

White lines were observed on the nails of more than one-third (34.6%) of the children exposed to the mine, but they were not seen for any of the unexposed children. The odds of white lines on nails are 12 times higher for exposed than that for unexposed children (OR = 12.11, p-value = 0.005) (Table 5).

#### Chronic colic

37.2% of the children exposed to the mine suffered from chronic colic whereas only 6.2% of the non-exposed children had a history of chronic colic. The odds of chronic colic are seven folds higher for exposed than that for non-exposed children (OR = 7.30, p-value = 0.007) (Table 5).

#### Dermatologic alterations

dermatologic alterations were detected in almost two-tenth (19.2%) of the children exposed to the mine but in none of the unexposed children. The observed dermatologic alterations included: *café-au-lait* spots in flexor areas of the abdomen, back, chest or neck; calluses on palms of the hands; soles with thick and rough skin; and calluses in areas without friction. The odds of dermatologic alterations are five times higher for exposed than that for non-exposed children (OR = 6.16, p-value = 0.013) (Table 5).

The presence of dermatological alterations significantly differed between male and female children exposed to the mine (p-value = 0.044). The odds of *café-au-lait* spots are higher in exposed males than in exposed females (OR = 4.18; p-value = 0.041).

#### Other clinical signs

We observed the following clinical signs in the study population: bluish line on the gum, calluses in body areas without friction, calluses on palms and sole with thick and rough skin, and inflamed conjunctiva. Children exposed to the mine have higher odds of developing these clinical signs than unexposed children (OR = 5.12, p-value = 0.024) (Table 5).

#### Altered mood

more than two-tenth (21.8%) of the exposed individuals showed an altered mood, i.e., irritability or depression, whereas none of the unexposed children presented these psychological symptoms. The odds of having an altered mood is seven times higher in children exposed to the mine than in unexposed children **(**OR = 7.07, p-value = 0.008) (Table 5).

## Discussion

We reported the presence of a chronic exposure to heavy metals in children exposed to mine in Cerro de Pasco and detected an association between those metals and several health conditions including history of nosebleed, chronic colic, dermatologic alterations, presence of white lines on nails, constipation, altered mood and reduced visual camp.

The exposure to heavy metal is chronic as children exposed to the open-pit mine continue to have high levels of heavy metals as reported in 2016^23^. The increased concentration of many heavy metals in hair root samples collected in 2018 as compared to that of 2016, and the presence of higher amount of heavy metals in hair tip than in hair root samples indicate an elevated diffusion of heavy metals in the environment from the expansion of mining activity in Peruvian Andes^17^. It should be noted that the Cerro mine was forced to build a wall in spring 2017 as a barrier to protect the inhabitants from heavy metal contamination, yet our findings reveal that the barrier is not a sufficient protective measure as high heavy metals concentrations were detected after the wall construction.

We showed a higher level of heavy metals in hair tip than in hair root samples of 2018. Hair tips are considered to be exposed for longer duration to heavy metals; between 12 and 28 months according to the speed of hair growth^32^.

The health effects of heavy metals depend on their concentration as well as on the co-exposure to other metals^33^. Therefore, although we showed that exposed children have high concentrations of most heavy metals and the latter is associated with several clinical symptoms, it was not possible to establish a clinical picture that characterizes each metal specifically. If the children had not presented high concentration of multiple heavy metals, it would have been easier to establish a relationship between the symptoms and a particular metal, since the health effects of each heavy metal is already known such as the case of lead (lead poisoning), mercury (Minamata disease) and arsenic (arsenicosis)^15,34^. It is also worthy to remember that long-term exposure to heavy metals is associated with the development of fatal diseases such as kidney failure, different types of cancer, and neuronal damage^1,3,13–16^.

We reported the presence of a higher concentration of heavy metals in boys than in girls exposed to the mine which could be related to cultural habits (family role and playing outside) that would give rise to gender difference in exposure to environmental agents.

Our findings about the presence of higher concentration of heavy metals in children living in the region exposed to the mine than in those living in the unexposed region is in line with that of previous studies about the presence of high heavy metals concentration in blood of Cerro de Pasco children as well as the contamination of the water and soil of the region by heavy metals^21,24,25^.

Our study has several limitations. The sample size of the unexposed group was small. Due to the design of our study, ecologic study design, the estimate heavy metal concentrations are proxy and not individual based as the rely on the average concentration of those metals in the population. The estimated measures of association (odds ratio) therefore were crude, i.e., not adjusted for potentially confounding variables. Despite these limitations, we believe that our study help point out the hazardous effects of mining activities without protective measures on the environment and health.

## Conclusions

The mining activities in Peru is a continuous threat to the environment and health of children living near the mine. They are associated with up to 15 folds increased risk of developing various health conditions. Actions should be taken to protect the environment and health.

## Supplementary Information

Supplementary Tables.

## Data Availability

All relevant data are included within the paper. Data contain potentially identifying information and sensitive participants information. For all these reasons, the authors must not upload the dataset to a stable, public repository. However, the authors agree to make freely available any materials and data described in the publication upon reasonable request to joseignacio.munoz.barus@usc.es, principal investigator of this project and professor at University of Santiago de Compostela.
