# Supplementary material for: Heavy metal contamination in Peru: implications on children’s health
Source: Sci Rep. 2021 Nov 23;11:22729. doi: 10.1038/s41598-021-02163-9 (PMC8611049; doi:10.1038/s41598-021-02163-9)
Supplement: Supplementary file 1 — Supplementary Tables. [file 41598_2021_2163_MOESM1_ESM.docx]

**Heavy metal contamination in Peru: implications on children´s health**

Xulia Fandiño Piñeiro^1^, Mauro T Ave^1,2,3,4,5^, Narmeen Mallah^5,6,7*^, Francisco Caamaño-Isorna^5,6,7^, A. Nuria Guisández Jiménez^1^, Duarte Nuno Vieira^8^, Flaviano Bianchini^9^, José Ignacio Muñoz-Barús^1,2^

^1^Department of Forensic Sciences, Pathology, Gynecology and Obstetrics, Pediatrics, University of Santiago de Compostela, Spain.

^2^Institute of Forensic Sciences, University of Santiago de Compostela, Spain

^3^Complejo Hospitalario Universitario de Santiago de Compostela, Santiago de Compostela. Spain.

^4^Centro de Investigación Biomédica en Red de Enfermedades Cardiovasculares (CIBERCV), Spain.

^5^Instituto de Investigación Sanitaria de Santiago (IDIS), Spain.

^6^Department of Preventive Medicine, University of Santiago de Compostela, 15782, Santiago de Compostela, Spain.

^7^Centro de Investigación Biomédica en Red de Epidemiología y Salud Pública (CIBERESP), Madrid, 28029, Spain.

^8^Department of Legal Medicine and Ethical Sciences. Faculty of Medicine, University of Coimbra, Portugal.

^9^Source International, Calci-Pisa, Italy

***Corresponding author:** Narmeen Mallah, Department of Preventive Medicine, University of Santiago de Compostela, Santiago de Compostela, Spain, R/ San Francisco, s/n, 15782, [[narmeen.mallah@usc.es](mailto:narmeen.mallah@usc.es)], +34-881-812-268.

**Table S1. Association between gender and heavy metal concentration across children living in Paragsha, region exposed to the mine.**

|  | **Average concentration (mg/Kg)** | |  |
| --- | --- | --- | --- |
| **Metal** | **Female  (N=38)** | **Male  (N=38)** | **P-value** |
| Cd | 0.07 | 0.13 | <0.05 |
| Cr | 0.69 | 0.98 |  |
| Pb | 2.80 | 6.32 |  |
| Cu | 15.86 | 11.13 |  |
| Sn | 28.97 | 55.03 |  |
| Co | 0.01 | 0.03 |  |

*Cd: cadmium; Co: cobalt; Cr: chromium; Pb: lead; Cu: copper; Sn: tin.*

**Table S2. Comparison of heavy metal concentrations between hair tip and hair root samples of the same children living in Paragsha, region exposed to the mine.**

| **Metal** | **Concentration (mg/Kg)** | | **T-test**  **P-value** |
| --- | --- | --- | --- |
|  | **Hair tip** | **Hair root** |  |
| Al | 49.64 | 32.07 | 0.001 |
| Ba | 4.84 | 1.46 | 0.001 |
| Be | 0.00 | 0.00 | 0.009 |
| Cd | 0.39 | 0.07 | <0.001 |
| Co | 0.14 | 0.03 | 0.002 |
| Cr | 1.50 | 0.69 | <0.001 |
| Fe | 77.36 | 53.21 | 0.033 |
| Mn | 13.21 | 3.48 | <0.001 |
| Mo | 0.18 | 0.09 | <0.001 |
| Pb | 13.08 | 3.51 | 0.005 |
| Cu | 23.86 | 12.47 | <0.001 |
| Sb | 0.24 | 0.11 | <0.001 |
| Sn | 65.44 | 26.15 | 0.001 |
| Tl | 0.02 | 0.01 | 0.009 |
| V | 0.24 | 0.06 | 0.008 |
| Zn | 407.86 | 202.93 | 0.001 |

*Al: aluminium; Ba: barium; Be: beryllium; Cd: cadmium; Co: cobalt; Cr: chromium; Fe: iron; Mn: magnesium; Mo: molybdenum; Pb: lead; Cu: copper; Sb: antimony; Sn: tin; Tl: thallium; V: vanadium; Zn: zinc.*

## **Table S3. T-student test of the association of heavy metals with history of nosebleed and the presence of white lines on the nails, irrespective of the place of residence.**

| **Metal** | **Nosebleed** | **N** | **Average**  **concentration**  **(mg/kg)** | **T-test p-value** | **White lines on the nails** | **N** | **Average**  **concentration**  **(mg/kg)** | **T-test p-value** |
| --- | --- | --- | --- | --- | --- | --- | --- | --- |
| Al | no | 42 | 25.70 | **0.031** | no | 66 | 26.31 | **0.002** |
|  | yes | 50 | 31.48 |  | yes | 26 | 35.27 |  |
| As | no | 42 | 0.35 | **0.015** | no | 66 | 0.39 | 0.102 |
|  | yes | 50 | 0.47 |  | yes | 26 | 0.49 |  |
| Cd | no | 42 | 0.06 | **0.003** | no | 66 | 0.08 | **0.014** |
|  | yes | 50 | 0.11 |  | yes | 26 | 0.12 |  |
| Cr | no | 42 | 0.04 | **0.043** | no | 66 | 0.71 | 0.092 |
|  | yes | 50 | 0.04 |  | yes | 26 | 0.88 |  |
| Fe | no | 42 | 42.64 | **0.007** | no | 66 | 46.50 | **0.016** |
|  | yes | 50 | 57.48 |  | yes | 26 | 61.38 |  |
| Mn | no | 42 | 3.64 | **0.035** | no | 66 | 4.12 | **0.047** |
|  | yes | 50 | 5.56 |  | yes | 26 | 6.12 |  |
| Pb | no | 42 | 2.70 | **0.034** | no | 66 | 3.01 | 0.055 |
|  | yes | 50 | 5.02 |  | yes | 26 | 6.38 |  |
| Sn | no | 42 | 24.54 | **0.023** | no | 66 | 31. 03 | 0.158 |
|  | yes | 50 | 43.26 |  | yes | 26 | 44.06 |  |
| Tl | no | 42 | 0.01 | **0.008** | no | 66 | 0.01 | **0.029** |
|  | yes | 50 | 0.02 |  | yes | 26 | 0.03 |  |
| V | no | 42 | 0.05 | 0.233 | no | 66 | 0.05 | **0.012** |
|  | yes | 50 | 0.02 |  | yes | 26 | 0.07 |  |

*N: number of children; Al: aluminium; As: arsenic; Cd: cadmium; Cr: chromium; Fe: iron; Mn: magnesium; Pb: lead; Sn: tin; Tl: thallium; V: vanadium.*
